# Evaluating the relative predictive validity of measures of self-referential processing for depressive symptom severity

**DOI:** 10.3389/fpsyt.2024.1463116

**Published:** 2025-02-10

**Authors:** Ethel Siew Ee Tan, Hong Ming Tan, Kah Vui Fong, Sheryl Yu Xuan Tey, Nikita Rane, Chong Wei Ho, Zhao Yuan Tan, Rachel Jing Min Ong, Chloe Teo, Jerall Yu, Maxine Lee, An Rae Teo, Sin Kee Ong, Xin Ying Lim, Jin Lin Kee, Jussi Keppo, Geoffrey Chern-Yee Tan

**Affiliations:** ^1^ Department of Mood and Anxiety, Institute of Mental Health, Singapore, Singapore; ^2^ National University of Singapore (NUS) Business School, National University of Singapore, Singapore, Singapore; ^3^ College of Humanities and Sciences, National University of Singapore, Singapore, Singapore; ^4^ Lee Kong Chian School of Medicine, Nanyang Technological University, Singapore, Singapore; ^5^ Faculty of Arts and Social Sciences, National University of Singapore, Singapore, Singapore; ^6^ Clinical Research Unit, National Healthcare Group (NHG) Polyclinics, Singapore, Singapore; ^7^ Ministry of Education, Singapore, Singapore; ^8^ Institute of Operations Research and Analytics, National University of Singapore, Singapore, Singapore

**Keywords:** self-schema, self-concept, self-referential processing, personality, depression

## Abstract

**Introduction:**

The self-referential encoding task (SRET) has a number of implicit measures which are associated with various facets of depression, including depressive symptoms. While some measures have proven robust in predicting depressive symptoms, their effectiveness can vary depending on the methodology used. Hence, understanding the relative contributions of population differences, word lists and calculation methods to these associations with depression, is crucial for translating the SRET into a clinical screening tool.

**Methods:**

This study systematically investigated the predictive accuracy of various SRET measures across different samples, including one clinical population matched with healthy controls and two university student populations, exposed to differing word lists. Participants completed the standard SRET and its variations, including Likert scales and matrix formats. Both standard and novel SRET measures were calculated and compared for their relative and incremental contribution to their associations with depression, with mean squared error (MSE) used as the primary metric for measuring predictive accuracy.

**Results:**

Results showed that most SRET measures significantly predicted depressive symptoms in clinical populations but not in healthy populations. Notably, models with task modifications, such as Matrix Endorsement Bias and Likert Endorsement Sum Bias, achieved the lowest mean squared error (MSE), indicating better predictive accuracy compared to standard Endorsement Bias measures.

**Discussion:**

These findings imply that task modifications such as utilising Likert-response options and the use of longer word lists may enhance the effectiveness of screening methods in both clinical and research settings, potentially improving early detection and intervention for depression.

## Introduction

Screening and monitoring of Depression have traditionally relied on self-reported measures of depressive symptoms, capturing the individual’s current mood but potentially overlooking underlying vulnerability or future risk (1). Self-referential processing (SRP), the processing of information related to one’s self, provides a measure of the individual’s perception of themselves, which is a core feature of depression (2–5). The self-referential encoding task (SRET) has been widely used to measure individuals’ self-schemas. In the SRET, an adjective is presented and participants are required to make binary decisions about whether the word describes themselves. Various measures including number of word endorsements, reaction time to decide, and number of words recalled are then collected to derive information regarding the participant’s emotional self-biases (6, 7).

Numerous studies have explored the associations between SRET measures and depression, including symptoms, longitudinal course, treatment response, relapse and remission (8–11). For instance, Dainer-Best et al. (12) found that depressed individuals tended to endorse more negative words and nondepressed individuals endorsed more positive words. Negative biases in SRP have also been linked to increased risk of recurrent depressive episodes (10), while deficits in SRP, such as slower drift rates in rejecting negative stimuli, have been shown to persist into remission among individuals with depression (8). Clustering approaches based on the SRET have also shown to be useful in subgrouping patients with depressive and anxiety symptoms, providing a novel transdiagnostic framework (13).

Nonetheless, several SRET variables have been inconsistent in their associations with depressive symptoms across different studies. A meta-analysis conducted on endorsement bias variables showed a mixed pattern of results (14): Some studies found that depressed individuals were more likely to endorse negative words (12), while others observed that depressed individuals had greater endorsement of positive words (9). On the other hand, Kiang et al. (15) found no significant differences in the endorsement of positive and negative words among depressed individuals. Nevertheless, those endorsing severe levels of depressive symptoms tended to endorse fewer positive words, suggesting a potential link with symptom severity.

This ambiguity in results extends to other SRET measures. Reaction time (RT), while initially shown to have no significant difference in clinically healthy and clinically depressed populations (16), was found to be slower in depressed individuals when processing self-referential adjectives (14, 17). In addition, several studies enhanced the interpretability of the RT data by incorporating drift rates in their analysis, revealing a significant correlation between mean drift rates for positive and negative words and baseline depression levels. Similarly, recall bias, initially shown to be significant in individuals with recurrent depressive episodes (10), showed no significant association with depressive symptom severity in a study by Dainer-Best et al. (12). These findings complicate the use of endorsement bias, RT and recall bias as reliable markers for depressive symptoms and underscores the complexity of assessing cognitive processes in MDD.

The inconsistencies between studies may be accounted for by population differences, procedural differences such as variations in the word lists used in the different studies and the method of calculating each variable across studies. For example, in Joorman et al.’s study (18) paper, endorsement was calculated by taking the number of words endorsed in a valence category divided by the total number of words endorsed while in LeMoult et al.’s study (10), endorsement was operationalised by taking the number of endorsed words in each valence category, thus introducing methodological inconsistencies.

There is a need to better understand the relative contributions of population differences, word lists and calculation methods to these associations with depression if the SRET is to be clinically translated. In this study, we aim to systematically investigate the predictive validity of the various SRET measures across different samples exposed to differing word lists. The primary aim is to evaluate the relative strength of association with depressive symptoms between SRET-based measures. To this end, we draw together data collected from clinical, community and healthy populations where participants completed a common self-report measure for depressive symptoms and a self-referential encoding task encompassing a range of measures. The self-referential encoding task includes a wide range of words encompassing common word lists from other studies such as LeMoult et al. (10), and Frewen and Lundberg (19). Each measure is then compared for its relative and incremental contribution to their associations with depression.

## Method

### Datasets and paradigms

In this study, three primary datasets were utilised for the comparative analysis:

The first dataset (Dataset A) comprised a Self-Referential Encoding Task (SRET) consisting of 60 words, incorporating endorsement data, latencies, recall data, recognition data as well as Matrix and Likert data for a separate set of 200 words. This task was administered to a sample of 188 participants, including 144 patients from the Institute of Mental Health (IMH) and 44 healthy controls. The clinical participants were recruited from IMH outpatient clinics if they had a history of depressive symptoms or were currently experiencing depressive symptoms, while the control group was recruited from the community. Details on the patients’ diagnostic status and other sample characteristics are provided in **Supplementary Table 1**.

Participants were drawn from three studies conducted at IMH, including 84 IMH patients from the “Understanding the person, exploring change across psychotherapies” (XChange) study, which also included 52 participants from the “Understanding the Person, Improving Psychotherapy: Preventing Relapse by targeting Emotional bias Modulation in PsychoTherapy” (PRE-EMPT) and 34 patients and 18 healthy controls from “The role of cholinergic dysfunction in the progression of depression” (CholDep) study. In the Choldep study, healthy controls were also recruited by word of mouth.

The second dataset (Dataset B) comprised another iteration of the SRET that consisted of 185 words, and has endorsement data, latencies and also components of an Other-Referential Processing (ORP) task. It was administered to 61 participants, who were recruited from the National University of Singapore (NUS) as part of an undergraduate thesis project.

The third dataset (Dataset C) also employed a SRET that consisted of 179 words and was administered to a separate sample of 97 participants, recruited at NUS for another undergraduate thesis project. This dataset included endorsement data, latencies and recall data.

### Measures

#### Self-referential encoding task (SRET)

The SRET is a task designed to access an individual’s self-relevant schemas (7) that typically comprises three sequential segments: endorsement, distractor task, and incidental recall.

In the endorsement task, participants judged whether an adjective described them. Participants responded by pressing keys representing “not me” or “me” on a computer keyboard. Reaction times, measured in milliseconds were also recorded.

Following the endorsement task, participants engaged in a five-minute distractor task to minimise interference and memory consolidation of endorsed words. Subsequently, only participants in sample A and C completed an incidental recall task lasting one minute, during which they were prompted to recall the words that have been displayed to them in the endorsement task. Afterward, participants in sample A were also presented with a list of 120 words and asked to indicate whether each word had been presented during the endorsement task. The list comprised 60 words from the endorsement task and 60 distractor words. Finally, sample A participants were presented with a matrix containing 200 words and asked to select which words described them. For each selected word, participants from Dataset A rated how accurately the word described them on a scale of 1-4 (1 = Not at all, 4 = Completely accurate).

The following measures derived from the SRET were calculated to evaluate the observed responses.

### Endorsement

Multiple endorsement variables were derived using varying calculation methods found in existing literature.

The Proportion of Negative Words Endorsed and Proportion of Positive Words Endorsed were computed as the number of positive/negative words endorsed divided by the number of positive/negative words presented, respectively.

Endorsement bias was operationalised as the number of positive/negative words endorsed divided by the total number of words endorsed. Additionally, a variable representing the difference between negative and positive biases was calculated by subtracting the positive endorsement bias from the negative endorsement bias.

### Latencies

Two reaction time (RT) variables were analysed. Positive RT bias and Negative RT bias were calculated to assess the differences in reaction times between endorsing and rejecting negative or positive words.

Positive RT bias was calculated by using the formula: Positive RT Bias = (Mean RT of Endorsement of Positive Words - Mean RT of Non-Endorsement of Positive Words)/Average RT Across all Trial Types. Similarly, Negative RT bias was calculated with the formula: Negative RT Bias = (Mean RT of Endorsement of Negative Words - Mean RT of Non-Endorsement of Negative Words)/Average RT Across all Trial Types.

This method of determining RT bias corresponds with the approach employed in previous SRET studies (20).

### Recall bias

Only words that were correctly recalled were considered. Negative Recall Bias was calculated by dividing the number of negative recalled and endorsed words by the total recalled and endorsed words. Additionally, the Proportion of Negative Endorsed and Recalled Words to Total Endorsed Words and the Proportion of Positive Endorsed and Recalled Words to Total Endorsed Words variables were also calculated.

### Recognition bias

Negative Recognition Bias was operationalised as the number of correctly recognised and endorsed negative words divided by total number of words recognised correctly while Positive Recognition Bias was defined as the number of correctly recognised and endorsed positive words divided by total number of words recognised correctly.

Signal Detection Theory (SDT) examines how individuals distinguish meaningful information from “noise” (21). The response bias metric, *c*, measures an individual’s tendency to respond affirmatively or negatively. In this study, *c* reflects the tendency of a particiapnt to respond whether or not they have seen the word in the word list of the preceding endorsement task. A value of *c* < 0 represents a strong bias towards “Yes”, while *c* > 0 represents a strong bias towards “No” (22). As such, we calculated the measures *c+*, *c-* and *c+* minus *c-* using the following formula:

Reference formula (23):


$$c=-\frac{\phi^{-1}\left(H\right)\;+\;\phi^{-1}\;\left(F\right)}{2}$$

$$*\text{H denotes hit rate }\left(\frac{number\;of\;correctly\;recognised\;words\;that\;were\;endorsed}{Total\;number\;of\;words\;in\;categorisation\;task}\right)$$

$$*\text{F denotes false alarm rate }(\frac{number\;of\;incorrectly\;recognised\;words\;that\;were\;endorsed}{Total\;number\;of\;words\;in\;categorisation\;task})$$

### Drift rate

Drift rates (*v*) for sample A were estimated using the drift diffusion model and it represents the speed and direction of information accumulation, with positive values suggesting a preference for the upper threshold (“Yes”). In the context of SRET, drift rate reflects the efficiency of processing whether negative or positive self-descriptive adjectives describe oneself.

Endorsement data and RT data underwent analysis using FAST-dm software, with parameters determined by the trial size of 60 words. Given the small sample size and expected presence of contaminated data, the Kolmogorov-Smirnov estimation test was chosen to run the program. Previous studies have also explored the association between SRET and depressive symptoms, incorporating drift rates as a measure (12, 24). Two drift rate measures were derived: 1) Drift rates towards negative words (*v-*), indicating the speed and direction of information accumulation when processing negative stimuli, and 2) Drift rates towards positive words (*v+*), indicating the speed and direction of information accumulation when processing positive stimuli.

### Matrix and Likert data

Similar to the endorsement measures, the Proportion of Matrix Negative Words Endorsed was calculated by taking the number of negative words endorsed in the matrix divided by the number of negative words presented in the matrix, and the same calculation was done for Proportion of Matrix Positive Words Endorsed. Another variable, Negative Matrix Endorsement Bias, was calculated by taking the number of negative words endorsed in the matrix divided by total number of words endorsed in the matrix.

For the Likert measures, the Proportion of Likert Sum Negative Words and Proportion of Likert Sum Positive Words were calculated by dividing the sum of the ratings of the negative or positive words endorsed by the total number of negative or positive words presented. The Negative Likert Endorsement Sum Bias was computed by dividing the sum of the ratings of negative words endorsed by the total number of non-zero responses, and the same method was applied to calculate the Positive Likert Endorsement Sum Bias.

Next, the Likert data underwent recoding: responses of 1 were adjusted to 0, while responses of 2 to 4 were recoded as 1. This was to avoid the assumption that there are equal intervals between scale points and to facilitate more intuitive interpretations of the results. Following recoding, the sum of negative and positive words was calculated. The same calculation was done for the Proportion of Likert Negative words and Proportion of Likert Positive words. Negative Likert Endorsement bias was computed by dividing the sum of negative words by the total number of non-zero responses, while Positive Likert Endorsement bias was calculated in a similar manner.

The mean rating of negative words endorsed on the Likert scale was calculated as the Mean Negative Likert Rating Bias. This was calculated by averaging the Likert ratings for all negative words endorsed and dividing by the total number of negative words presented.

### Overlapping words

As each of the three datasets utilised a unique set of word lists, with overlaps in words between them, we conducted regressions using only the overlapping words to investigate the influence of participant characteristics versus word lists on predictive differences. Across all three datasets, we identified a total of 23 overlapping words.

### Depressive symptoms

The Quick Inventory of Depressive Symptomatology (QIDS-16-SR) is a 16-item self-report measure that assesses the 9 criterion domains used to diagnose a major depressive disorder (25) and was a shortened version derived from the Inventory of Depressive Symptomatology (IDS). Participants were asked to rate the severity of each of the 30 symptoms in the preceding seven days on a scale of 0–3, with higher scores indicating greater symptom severity. The total score is calculated by summing 9 of the 30 items. The total score ranges from 0 to 27. Participants were categorised into depressive symptom severity levels based on their total QIDS-16-SR scores: 0–5 (no depression), 6–10 (mild depression), 11–15 (moderate depression), 16–20 (severe depression), and 21–27 (very severe depression).

QIDS-16-SR was shown to have satisfactory psychometric properties: Cronbach’s alpha was.875 for Dataset A,.774 for Dataset B and.837 for Dataset C, displaying good reliability in the current study. QIDS-SR-16 total scores were also highly correlated with IDS-30-SR (*r* = .96) and HAM-D (*r* = .86) total scores. Overall, the QIDS-16-SR exhibited excellent psychometric properties, suggesting its utility as a brief assessment tool for depressive symptom severity in both clinical and research settings (25).

### Multiple linear regression analyses

We conducted multiple linear regression analyses to examine the associations between the different SRET variables such as negative/positive endorsement bias, negative/positive latency bias with depressive symptoms, while controlling for demographic variables. Every SRET variable was entered as independent variables separately in regression models. Demographic variables, including age, gender, and education level, were included as covariates in the models to account for potential confounding effects.

Regression analyses were performed separately for each dataset (Dataset A, Dataset B, and Dataset C) to explore potential variations in the associations across different samples. R^2^ values of the regression models will be utilised as a measure to evaluate the relative strength of associations between the SRET variables and depressive symptoms.

### Mean squared error as primary metric

Mean square error (MSE) is measured as the average of the squared differences between each of the actual values and the predicted values by a model. The formula for calculating MSE is as follows:

$MSE=\frac{\sum_{i=1}^{n}{\left(y_{i}-p_{i}\right)}^{2}}{n}$ , where y_i_ is the i^th^ instance of the actual value and p_i_ is the predicted value, and n is the total number of values (26).

For regression tasks, MSE evaluates how well the actual data is represented by a model’s predictions, with lower MSE values indicating better model performance. Hence, MSE values were used to select the models as they measure the prediction error of SRET measures (27). In this context, the model with a lower MSE value would indicate a better fit in determining the severity of depressive symptoms.

### R^2^ as metric

R^2^ quantifies the proportion of variance in depressive symptoms that is explained by the different SRET measures. Higher R^2^ values indicate that a larger proportion of the variance in depressive symptoms can be accounted for by the SRET measures, suggesting stronger associations and provision of better fit, hence allowing for a comparison of the relative predictive power of the SRET variables in explaining variations in depressive symptoms (28). However, as Ford (29) highlighted, R^2^ can be arbitrarily low even with a well-fitted model and arbitrarily close to 1 with a flawed model, underscoring the decision to rely on additional metrics like MSE to examine predictive ability.

All analyses were conducted using R (30) on RStudio version 12.0.353.

## Results

### Demographics

In this study, we compared the SRET variables between three datasets: a clinical population with matched healthy controls (Dataset A) and two university populations (Datasets B and C) using R^2^ values of endorsement, RT and recall. QIDS-16-SR Symptom Severity was calculated for the participants of each dataset. In Dataset A, the breakdown of depression severity was analysed separately for the patient (N = 144) and healthy control (N = 44) samples. In the patient sample, 6.94% presented with no depression, 24.31% with mild depression, 36.81% with moderate depression, 20.83% with severe depression and 7.64% with very severe depression. In contrast, the healthy control sample had 54.55% of the participants presenting with no depression, 29.55% with mild depression, 4.55% with moderate depression, 2.27% with severe depression and no healthy controls were categorised as having very severe depression. There were 9 participants in Dataset A with missing data. In Dataset B (N = 61), 32.79% reported no depression, 49.18% reported mild depression, 9.84% reported moderate depression and 8.20% reported severe depression. In Dataset C (N = 97), there were 3 participants with missing QIDS-16-SR data. 49.48% of the participants reported no depression, 25.77% reported mild depression, 19.59% reported moderate depression, 1.03% reported severe depression and 1.03% reported very severe depression. The demographic characteristics of participants of each dataset are presented in **Supplementary Table 1**.

### Comparison of SRET variables between datasets

Regression analyses showed that endorsement bias variables and RT bias variables were significantly associated with depressive symptoms only for Dataset A, while no significant associations were found in datasets B and C, representing the non-clinical population. Please refer to **Supplementary Tables 2**, **3** for the regression models of the effect of endorsement bias and RT bias for full word list on depressive symptoms, across all three datasets and **Supplementary Table 4** for the comparison of effects of recall bias variables for Datasets A and C.

This also held true when only calculating measures from the 23 overlapping words between the datasets. Regression analysis conducted on the 23 overlapping words between datasets revealed that the regression models were statistically non-significant when Dataset B and C were combined. Similarly, the models for all seven predictors of Endorsement and RT bias were statistically non-significant for Dataset B and Dataset C. Only the regression models in Dataset A were statistically significant, both when the data were analysed separately for the Patient and Healthy Control subgroups, and when the total sample was analysed. Please refer to **Supplementary Tables 5**, **6** for the regression models of the effect of endorsement measures and RT measures on depressive symptoms for the 23 overlapping words, across all populations.

In Dataset A alone, regression analysis on the 23 overlapping words showed that Proportion of Negative Words Endorsed had an MSE value of 22.01 (*F*(8, 166) = 16.73, *p* <.001, R^2^ = 0.414) and Negative Endorsement Bias with an MSE value of 22.73 (*F*(8, 166) = 15.44, *p* <.001, R^2^ = 0.394). These two models had the lowest and comparable MSE values. This was followed by the Difference in Endorsement Bias variable with an MSE value of 25.46 (*F*(8, 159) = 9.36, *p* <.001, R^2^ = 0.292), Positive Endorsement Bias with an MSE value of 25.62 (*F*(8, 159) = 9.16, *p* <.001, R^2^ = 0.287) and lastly, Proportion of Positive Words Endorsed with an MSE value of 27.10 (*F*(8, 166) = 9.13, *p* <.001, R^2^ = 0.278). Comparatively, the RT bias variables performed worse than endorsement bias variables, with both variables yielding non-significant p-values. These findings suggest that endorsement bias variables displayed stronger predictive power compared to RT bias variables in Dataset A. Please refer to **Supplementary Table 7** for the full regression models.

To investigate the consistent lack of significance observed in Datasets B and C, we conducted an F-test comparing the variance in depressive symptoms between Dataset A and the combined Datasets B and C level of significance and there appeared to be a significant trend towards greater variance in depressive symptoms in Dataset A (*F*(183, 157) *=*1.75, *p* <.001). Subsequent analyses were conducted on the full word list in Dataset A alone as it demonstrated more robust associations with depressive symptoms.

### Endorsement

The model with the Negative Endorsement Bias as a predictor had a MSE value of 20.76 and R^2^ value of 0.447 (*F*(8, 166) = 19.17, *p* <.001) in Dataset A. Overall, Negative Endorsement Bias ranked 7^th^ in its MSE values among the other predictors. On the other hand, the model with Positive Endorsement Bias as a predictor had a MSE value of 33.60 and R^2^ value of 0.105 (*F*(8, 166) = 2.78, *p* <.001) and the model with the Difference in Endorsement Bias as a predictor had a MSE value of 29.65 and R^2^ value of 0.210 (*F*(8, 166) = 6.30, *p* <.001). These measures ranked 28^th^ and 24^th^ respectively. The Proportion of Negative Words Endorsed and Proportion of Positive Words Endorsed had a MSE of 22.06 (*F*(8, 166) = 16.63, *p* <.001, R^2^ = 0.412) and 24.92 (*F*(8, 166) = 12.00, *p* <.001, R^2^ = 0.336) respectively, ranking 10^th^ and 15^th^ among the SRET predictors for Dataset A.

The model with Negative RT Bias had a MSE value of 29.41 and a significant R^2^ of 0.216 (*F*(8, 166) = 6.55, *p <*.001) in Dataset A. Positive RT bias had a slightly higher MSE value of 29.44 and a significant R^2^ of 0.216 (*F*(8, 166) = 6.52, *p <*.001) in Dataset A. However, the MSE values for RT biases are outperformed by other predictors such as Negative Endorsement Bias and Matrix and Likert endorsement bias. This suggests that the endorsement bias predictors provide a better fit in predicting depressive symptoms. Overall, Positive and Negative RT Bias ranked 23^rd^ and 22nd respectively in terms of MSE values among the other SRET predictors.

Conversely, the Proportion of Negative Endorsed and Recalled Words to Total Endorsed Words model had a higher MSE value than RT Bias variables of 31.77 and a significant R^2^ of 0.154 (*F*(8, 166) = 4.30, *p <*.001). Similarly for the model with ​​Proportion of Positive Endorsed and Recalled Words to Total Endorsed Words as a predictor, it had a higher MSE value of 33.83 but a lower R^2^ of 0.099 (*F*(8, 166) = 2.59, *p <*.001). Comparatively, Negative Recall Bias had a lower MSE value of 28.39 and a higher R^2^ value of 0.241 (*F*(8, 148) = 6.72, *p <*.001). Similarly to RT Bias, the MSE values of Recall Bias are outperformed by the Endorsement Bias predictors. Overall, Proportion of Negative Endorsed and Recalled Words to Total Endorsed Words model ranked 26^th^, Proportion of Positive Endorsed and Recalled Words to Total Endorsed Words ranked 29^th^ and Negative Recall Bias ranked 20^th^. The Proportion of Positive Endorsed and Recalled Words to Total Endorsed Words ranked the lowest out of all SRET predictors.

### Recognition bias and drift rates

The model with Negative Recognition Bias had a MSE value of 22.50 (*F* (8, 165) = 15.84, *p* <.001, R^2^ = 0.402), while the model with Positive Recognition Bias yielded an MSE of 26.48 (*F* (8, 165) = 9.91, *p* <.001, R^2^ = 0.296). The models rank 12^th^ and 18^th^ respectively.

Response biases for recognition of endorsed negative words (*c-*) and recognition of endorsed positive words (*c+*) yielded a MSE value of 29.94 (*F* (8, 162) = 5.81, *p* <.001, R^2^ = 0.202) and 32.10 (*F* (8, 161) = 3.87, *p* <.001, R^2^ = 0.144) respectively. Notably, the Difference between the two response Biases (*c+ minus c-*) model yielded the lowest MSE value of 26.13 (*F* (8, 161) = 10.01, *p* <.001, R^2^ = 0.303) of the SDT measures and ranked 17^th^ followed by Response Bias for Recognition of Endorsed Negative Words (*c-*) at 25^th^ and Response Bias for Recognition of Endorsed Positive Words (*c+*) that ranked 27^th^ among the other SRET predictors.

Furthermore, drift rates towards negative words and positive words were analysed in Dataset A to understand their relationship with depressive symptoms. Drift rate towards Negative words (*v-*) yielded a MSE value of 28.72 (*F* (8, 162) = 6.90, *p* <.001, R^2^ = 0.230). Drift Rate towards positive words (*v+*) had a lower MSE value of 27.32 (*F* (8, 162) = 8.44, *p* <.001, R^2^ = 0.267). The MSE value suggests that Drift Rate towards positive words (*v+*) provides a slightly better fit for predicting depressive symptoms, compared to Drift rate towards negative words (*v-*). However, overall, the model with Drift Rate towards positive words (*v+*) ranked 19^th^ among the other SRET predictors.

### Endorsement bias using Matrix format and Likert scale

Negative Matrix Endorsement Bias in Dataset A reported a MSE value of 17.70 and a R^2^ value of 0.528 (*F*(8, 166) = 26.58, *p* <.001). The Negative Matrix Endorsement Bias model has the second lowest MSE value among all the models tested on Dataset A. The Proportion of Matrix Negative Words Endorsed reported a higher MSE value of 21.96 and a R^2^ of 0.415 (*F*(8, 166) = 16.82, *p* <.001) and the Proportion of Matrix Positive Words Endorsed reported an even higher MSE value of 24.74 and R^2^ of 0.341 (*F*(8, 166) = 12.26, *p* <.001). Overall, the Proportion of Matrix words endorsed variables ranked 9^th^ and 14^th^ respectively. This suggests that the Negative Matrix Endorsement Bias model is a good fit and is one of the best models for predicting depressive symptoms.

Difference in Likert Endorsement Sum Bias ranked first among the SRET predictors with the lowest MSE of 17.51 and the highest R^2^ value of 0.533 (*F* (8, 166) = 27.11, *p* <.001), suggesting that it is the best fit model for predicting depressive symptoms in Dataset A. Comparatively, Difference in Likert Endorsement Bias ranked 5^th^ among the SRET predictors with a MSE of 18.40 (*F* (8, 166) = 24.66, *p* <.001, R^2^ = 0.510). Taken together, these results suggest that Difference in Likert Endorsement Sum Bias, obtained using the sum of Likert responses, is a better predictor than the Difference in Likert Endorsement Bias, where the Likert responses were recoded into a binary variable.

**Figure 1:**
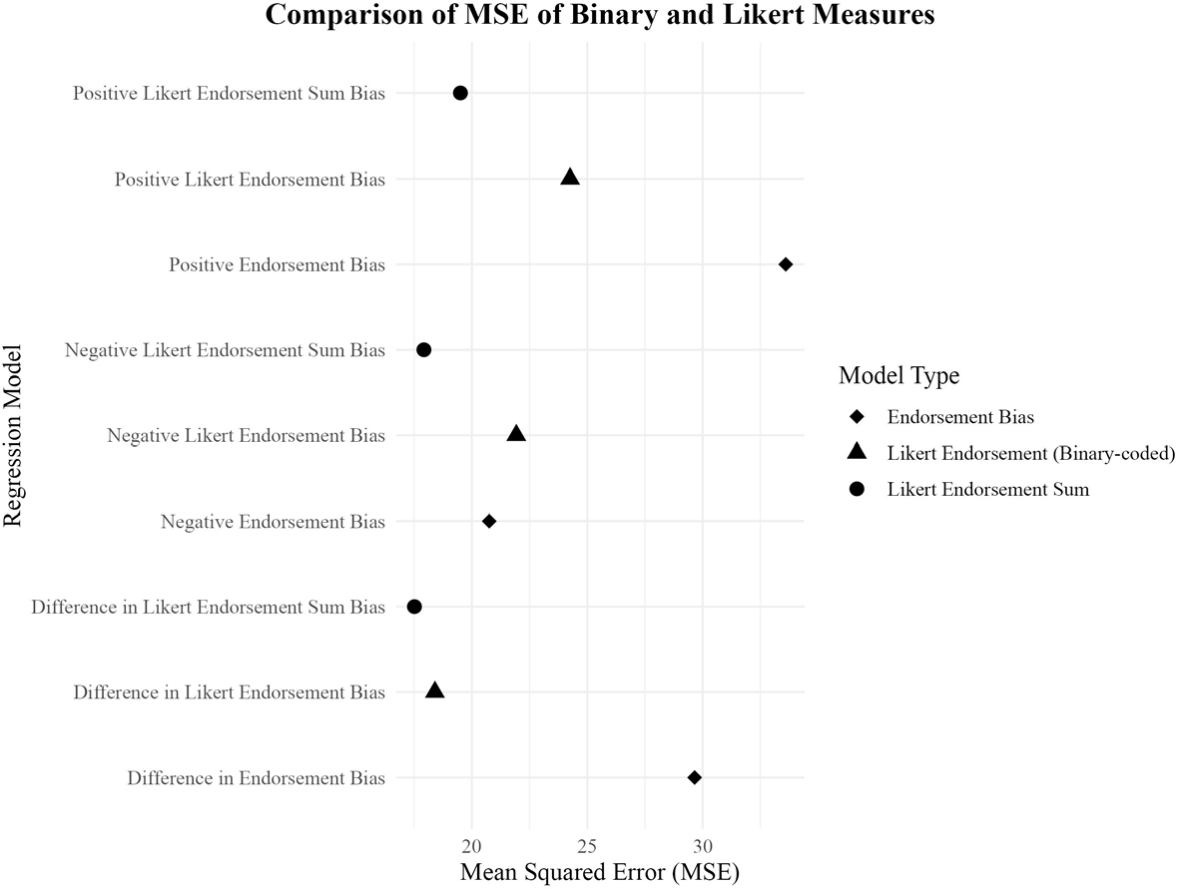
Scatter plot comparing MSE values of binary and Likert measures for predicting depressive symptoms.

The Negative Likert Endorsement Sum Bias and Positive Likert Endorsement Sum Bias yielded MSE values of 17.92 (*F* (8, 166) = 25.94, *p* <.001, R^2^ = 0.522) and 19.51 (*F* (8, 166) = 21.91, *p* <.001, R^2^ = 0.480) respectively, ranking 4^th^ and 6^th^ among the predictors. In comparison, the Negative Likert Endorsement Bias and Positive Likert Endorsement Bias reported MSE values of 21.93 (*F* (8, 166) = 16.88, *p* <.001, R^2^ = 0.416) and 24.26 (*F* (8, 166) = 12.98, *p* <.001, R^2^ = 0.354) respectively, ranking 8^th^ and 13^th^ among all predictors. When comparing the valenced measures, Negative measures yielded lower MSE values compared to Positive measures. These results also follow the same pattern as the Difference scores, where Likert Endorsement Sum Bias reported lower MSEs compared to Likert Endorsement Bias, with both Likert Scale measures yielding lower MSEs compared to regular and Matrix format. **Figure 1** shows the comparison of MSE values between Likert-response variables and binary-coded or binary-response variables.

Proportion of Likert Negative Words Endorsed ranked 3^rd^ among the SRET predictors with a MSE of 17.78 and R^2^ value of 0.526 (*F* (8, 166) = 26.35, p <.001), suggesting that the model is a good fit for predicting depressive symptoms in Dataset A. The Mean Negative Likert Bias reported a MSE of 16.50 and R^2^ of 0.410 (*F* (8, 166) = 16.50, p <.001), ranking 11^th^ overall. The Proportion of Likert Sum Negative Words reported a MSE value of 22.13 (*F* (8, 166) = 16.50, p <.001, R^2^ = 0.410) and the Proportion of Likert Sum Positive Words reported a MSE value of 25.19 (*F* (8, 166) = 11.62, p <.001, R^2^ = 0.329), ranking 11^th^ and 16^th^ among the SRET variables respectively.

Overall, the MSE values reported from all the models tested in Dataset A are consistent with the R^2^ values, indicating which models provide the best fit for predicting depressive symptoms. The model with the lowest MSE value was the Difference in Likert Endorsement Sum Bias, followed by Negative Matrix Endorsement Bias and the Proportion of Likert Negative Words Endorsed. Negative Likert Endorsement Sum Bias ranked fourth and the Difference in Likert Endorsement Bias ranked fifth in terms of lowest MSE values.

Conversely, the models with the highest MSE values included Proportion of Positive Endorsed and Recalled Words to Total Endorsed Words, Positive Endorsement Bias and Response Bias for Recognition of Endorsed Positive Words (c+). These models indicated a poorer fit for predicting depressive symptoms compared to those with lower MSE values.

All MSE rankings of each model are noted down in **Table 1**.

**Table 1:** Regression analysis of endorsement bias for full word list with depressive symptoms for Dataset A.

Variable	*B*	*SE*	95% CI		*t*	*p*	Model			
			LL	UL			R^2^	MSE	*F(df)*	*p*
Endorsement Bias										
Proportion of Negative Words Endorsed (MSE rank: 10)	13.60	1.36	10.91	16.29	9.98	1.11E^-18^	0.412	22.06	16.63 (8, 166)	8.16E^-20^
Proportion of Positive Words Endorsed (MSE rank: 15)	-12.48	1.50	-15.45	-9.52	-8.31	3.09E^-14^	0.336	24.92	12.00 (8, 166)	1.32E^-14^
Negative Endorsement Bias (MSE rank: 7)	15.71	1.45	12.84	18.58	10.78	6.70E^-21^	0.447	20.76	19.17 (8, 166)	5.77E^-22^
Positive Endorsement Bias (MSE rank: 28)	-2.29	0.79	-3.86	-0.74	-2.90	4.16E^-3^	0.105	33.60	2.78 (8, 166)	8.76E^-3^
Difference in Endorsement Bias (MSE rank: 24)	3.38	0.60	2.20	4.58	5.62	7.61E^-8^	0.210	29.65	6.30 (8, 166)	1.13E^-7^
RT Bias										
Positive RT Bias (MSE rank: 23)	-6545.39	1138.08	-8792.37	-4298.41	-5.75	4.16E^-8^	0.216	29.44	6.52 (8, 166)	5.49E^-8^
Negative RT Bias (MSE rank: 22)	5497.89	953.00	3616.32	7379.46	5.76	3.81E^-8^	0.216	29.41	6.55 (8, 166)	3.80E^-9^
Drift Rates										
Drift rates to Negative words (*v-*) (MSE rank: 21)	2.83	0.47	1.89	3.78	5.92	1.80E^-8^	0.230	28.72	6.90 (8, 162)	2.56E^-9^
Drift rates to Positive words (*v+*) (MSE rank: 19)	-3.30	0.49	-4.27	-2.33	-6.72	2.86E^-10^	0.267	27.32	8.44 (8, 162)	1.10E^-10^
Recall Bias										
Proportion of Negative Endorsed and Recalled Words to Total Endorsed Words (MSE rank: 26)	18.96	4.41	10.26	27.67	4.30	4.58E^-5^	0.154	31.77	4.30 (8, 166)	4.58E^-5^
Proportion of Positive Endorsed and Recalled Words to Total Endorsed Words (MSE rank: 29)	-10.00	3.72	-17.34	-2.66	-2.691	7.68E^-3^	0.099	33.83	2.59 (8, 166)	2.52E^-3^
Negative Recall Bias (MSE rank: 20)	8.57	1.37	5.87	11.29	6.25	4.09E^-9^	0.241	28.39	6.72 (8, 148)	1.03E^-9^
Recognition Bias										
Negative Recognition Bias (MSE rank: 12)	18.85	1.93	15.03	22.68	9.73	5.67E^-18^	0.402	22.50	15.84 (8, 165)	1.13E^-18^
Positive Recognition Bias (MSE rank: 18)	-16.29	2.18	-20.60	-11.98	-7.46	4.67E^-12^	0.296	26.48	9.91 (8, 165)	2.26E^-12^
Response Bias for Recognition of Endorsed Positive Words (*c+*) (MSE rank: 27)	1.90	0.49	0.94	2.86	3.91	1.37E^-4^	0.144	32.10	3.87 (8, 161)	6.38E^-5^
Response Bias for Recognition of Endorsed Negative Words (*c-*) (MSE rank: 25)	-2.81	0.53	-3.86	-1.76	-5.29	3.89E^-7^	0.202	29.94	5.81 (8, 162)	1.49E^-6^
*c+ minus c-* (MSE rank: 17)	2.61	0.35	1.92	3.30	7.46	5.21E^-12^	0.303	26.13	10.01 (8, 161)	1.69E^-12^
Matrix Endorsement Bias										
Proportion of Matrix Negative Words Endorsed (MSE rank: 9)	18.25	1.81	14.67	21.85	10.04	7.44E^-19^	0.415	21.96	16.82 (8, 166)	2.46E^-19^
Proportion of Matrix Positive Words Endorsed (MSE rank: 14)	-13.83	1.64	-17.08	-10.59	-8.42	1.68E^-14^	0.341	24.74	12.26 (8, 166)	1.83E^-15^
Negative Matrix Endorsement Bias (MSE rank: 2)	15.83	1.23	13.40	18.27	12.85	1.11E^-26^	0.528	17.70	26.58 (8, 166)	7.51E^-28^
Likert Endorsement Bias										
Proportion of Likert Sum Negative Words (MSE rank: 11)	7.22	0.72	5.79	8.66	9.94	1.46E^-18^	0.410	22.13	16.50 (8, 166)	2.56E^-19^
Proportion of Likert Sum Positive Words (MSE rank: 16)	-5.47	0.67	-6.80	-4.15	-8.16	7.64E^-14^	0.329	25.19	11.62 (8, 166)	2.41E^-14^
Positive Likert Endorsement Sum Bias (MSE rank: 6)	-6.05	0.52	-7.09	-5.02	-11.59	3.74E^-23^	0.480	19.51	21.91 (8, 166)	1.06E^-23^
Negative Likert Endorsement Sum Bias (MSE rank: 4)	6.12	0.48	5.17	7.08	12.68	3.18E^-26^	0.522	17.92	25.94 (8, 166)	1.23E^-27^
Difference in Likert Endorsement Sum Bias (MSE rank: 1)	3.26	0.25	2.77	3.76	12.98	4.61E^-27^	0.533	17.51	27.11 (8, 166)	2.89E^-28^
Proportion of Likert Negative Words Endorsed (MSE rank: 3)	14.33	1.12	12.12	16.54	12.79	1.60E^-26^	0.526	17.78	26.35 (8, 166)	1.20E^-27^
Positive Likert Endorsement Bias (MSE rank: 13)	-15.05	1.73	-18.47	-11.63	-8.69	3.20E^-15^	0.354	24.26	12.98 (8, 166)	5.46E^-16^
Negative Likert Endorsement Bias (MSE rank: 8)	19.85	1.97	15.96	23.75	10.06	6.68E^-19^	0.416	21.93	16.88 (8, 166)	1.49E^-19^
Difference in Likert Endorsement Bias (MSE rank: 5)	12.00	0.97	10.09	13.93	12.35	2.85E^-25^	0.510	18.40	24.66 (8, 166)	3.23E^-26^
Mean Negative Likert Bias (MSE rank: 11)	7.22	0.72	5.79	8.66	9.94	1.46E^-18^	0.410	22.13	16.50 (8, 166)	2.56E^-19^

This table presents the results of regression analysis for SRET measures on depressive symptoms. Standard errors were derived from the regression analysis conducted using RStudio. Statistical significance was determined using *p <.05.* However, for comparing R^2^ values, the exact values were reported. These measures include positive endorsement bias and negative endorsement bias, positive matrix endorsement bias and negative matrix endorsement bias. Please refer to the Methods section for details on how these measures were calculated.

### Comparison of SRET measures in dataset A

The full correlation matrix between Age, QIDS-16-SR scores, and all SRET measures in Dataset A is provided in **Supplementary Table 8**. For clarity, only the correlations for the best-performing variables within each group are highlighted here. Negative Endorsement Bias showed a strong correlation with Negative Matrix Endorsement Bias (*r* = 0.88, *p* <.001), and Negative Matrix Endorsement Bias was most strongly correlated with the Difference in Likert Endorsement Sum (*r* = 0.98, *p* <.001). Additionally, Negative Recall Bias was also highly correlated with Difference in Likert Endorsement Sum (*r* = 0.66, *p* <.001). Negative RT Bias was highly correlated with Negative Likert Endorsement Bias (*r* = 0.58, *p* <.001) while drift rates to positive words (*v+*) was the most highly correlated with Positive RT Bias (*r* = 0.66, *p* <.001).

### Incremental predictive validity

Given that the endorsement variables are among the most commonly used and strongest predictors of depressive symptoms in the SRET literature, the count of negative and positive words endorsed were entered as predictors in the base regression model. This base regression model yielded a MSE value of 21.92 (*F*(2, 149) = 52.28, *p* <.001) and a R^2^ value of 0.412. Subsequently, each of the remaining variables were individually added to the base regression model, and the change in the MSE values was examined to assess whether additional variables offer incremental predictive validity beyond the count of endorsed words.

The results show that the Negative Matrix Endorsement Bias (Δ MSE = -3.28, Δ R^2^ = 0.088) and Likert Endorsement Bias variables (Proportion of Likert Negative Words Endorsed (Δ MSE = -3.11, Δ R^2^ = 0.083), Difference in Likert Endorsement Sum Bias (Δ MSE = -3.01, Δ R^2^ = 0.081), Negative Likert Endorsement Sum Bias (Δ MSE = 2.70, Δ R^2^ = 0.072), Difference in Likert Endorsement Bias (Δ MSE = -2.28, Δ R^2^ = 0.061), Negative Likert Endorsement Bias (Δ MSE =-1.76, Δ R^2^ = 0.047)) showed the most significant changes in MSE values from the base regression model. A greater decrease in MSE values upon adding each measure to the baseline model suggests that these measures provide substantial improvements in predicting depressive symptoms. Specifically, Negative Matrix Endorsement Bias improved the model’s performance, resulting in a reduction of the MSE value by 3.28 compared to the baseline model. In contrast, the addition of Negative Recall Bias and Positive Recognition Bias to the baseline model did not yield improvements in predictive ability of the model, but instead reported an opposite trend where MSE increased by 0.07 and 0.01 respectively. The corresponding change in R^2^ was minimal at 0.002, 0.001 and 0.001 respectively. Positive RT Bias, Positive Endorsement Bias, Response Bias for Recognition of Endorsed Positive Words (c+) as well as the simple proportions of negative and positive words endorsed did not yield significant improvements in predictive ability, reporting zero change in MSE and R^2^, suggesting that none of these predictors significantly enhanced the model beyond the total count of endorsed words.

Please refer to **Table 2** for the MSE values as predictors are iteratively added individually to the base model, and the corresponding change in MSE is observed.

**Table 2:** Iterative addition of predictors to base regression model and corresponding R^2^ values.

Predictor	R^2^	ΔR^2^	MSE	ΔMSE	*p*
Negative Matrix Endorsement Bias	0.500	0.088	18.64	-3.28	3.56E^-22^
Proportion of Likert Negative Words Endorsed	0.496	0.083	18.81	-3.11	6.91E^-22^
Difference in Likert Endorsement Sum Bias	0.493	0.081	18.91	-3.01	1.01E^-21^
Negative Likert Endorsement Sum Bias	0.485	0.072	19.21	-2.70	3.29E^-21^
Difference in Likert Endorsement Bias	0.473	0.061	19.64	-2.28	1.64E^-20^
Negative Likert Endorsement Bias	0.460	0.047	20.16	-1.76	1.12E^-19^
Proportion of Matrix Negative Words Endorsed	0.459	0.047	20.17	-1.75	1.17E^-19^
Proportion of Likert Sum Negative Words	0.456	0.043	20.30	-1.62	1.86E^-19^
Mean Negative Likert Bias	0.456	0.043	20.30	-1.62	1.86E^-19^
Positive Likert Endorsement Sum Bias	0.456	0.043	20.31	-1.61	1.92E^-19^
Proportion of Negative Endorsed and Recalled Words to Total Endorsed Words	0.426	0.014	21.40	-0.52	8.89E^-18^
Drift rates to Negative words (*v-*)	0.424	0.011	21.49	-0.43	1.22E^-17^
Positive Likert Endorsement Bias	0.424	0.011	21.50	-0.42	1.27E^-17^
Proportion of Matrix Positive Words Endorsed	0.421	0.009	21.58	-0.34	1.65E^-17^
Negative Recognition Bias	0.421	0.008	21.61	-0.30	1.87E^-17^
Proportion of Likert Sum Positive Words	0.419	0.006	21.68	-0.23	2.36E^-17^
Drift rates to Positive words (*v+*)	0.417	0.004	21.75	-0.16	2.99E^-17^
Negative RT Bias	0.416	0.004	21.77	-0.15	3.17E^-17^
Negative Endorsement Bias	0.416	0.004	21.77	-0.14	3.20E^-17^
Proportion of Positive Endorsed and Recalled Words to Total Endorsed Words	0.415	0.003	21.81	-0.10	3.65E^-17^
Negative Recall Bias	0.414	0.002	21.85	0.07	4.16E^-17^
*c+* minus *c-*	0.413	0	21.90	-0.02	4.89E^-17^
Response Bias for Recognition of Endorsed Negative Words (*c-*)	0.413	0	21.90	-0.02	4.89E^-17^
Positive Recognition Bias	0.413	0	21.91	0.01	5.02E^-17^
Positive RT Bias	0.412	0	21.91	0	5.11E^-17^
Response bias for Recognition of Endorsed Positive Words (*c+*)	0.412	0	21.92	0	5.17E^-17^
Difference in Endorsement Bias	0.412	0	21.92	0	5.16E^-17^
Positive Endorsement Bias	0.412	0	21.92	0	5.16E^-17^
Proportion of Negative Words Endorsed	0.412	0	21.92	0	6.27E^-17^
Proportion of Positive Words Endorsed	0.412	0	21.92	0	6.27E^-17^

This table presents the R^2^ values and MSE as predictors are iteratively added individually to the base model, sorted from highest to lowest change in R^2^ and MSE This provides insight into the corresponding change in R^2^ and MSE observed with the addition of each predictor.

### Most influential subset of variables

Considering the wide range of predictors in our dataset, it was valuable to identify a subset of variables that best explain the variability in depressive symptoms. To achieve this, we employed both forward step regression and backward step regression techniques. These methods iteratively add or remove predictors based on their contribution to the model’s predictive power.

Four models were generated to identify the optimal subset of variables using different criterions: (1) Forward Stepwise Regression with AIC as criterion, (2) Backward Stepwise Regression with AIC as criterion, (3) Forward Stepwise Regression with BIC as criterion and (4) Backward Stepwise Regression with BIC as criterion.

The selected models of each method are presented in **Table 3**.

**Table 3:** Final Backward Stepwise Regression and Forward Stepwise Regression Models.

Model	Final Variables	AIC	BIC	R^2^	MSE
(1) Forward Stepwise Regression (AIC)	Negative Matrix Endorsement Bias, Negative Likert Endorsement Bias, Drift rates to Negative words (*v-*), Proportion of Negative Endorsed and Recalled Words to Total Endorsed Words	879.13	897.28	0.53	17.58
(2) Backward Stepwise Regression with (AIC)	Negative Matrix Endorsement Bias, Negative Likert Endorsement Bias	880.06	913.33	0.56	16.56
(3) Forward Stepwise Regression with (BIC)	Proportion of Negative Endorsed and Recalled Words to Total Endorsed Words, Proportion of Positive Endorsed and Recalled Words to Total Endorsed Words, Negative Recall Bias, Drift rates to Negative words (*v-*), Negative Matrix Endorsement Bias, Proportion of Matrix Positive Words Endorsed, Negative Likert Endorsement Sum Bias, Positive Likert Endorsement Sum Bias, Proportion of Likert Sum Positive Words	879.82	891.92	0.51	18.13
(4) Backward Stepwise Regression with (BIC)	Proportion of Negative Endorsed and Recalled Words to Total Endorsed Words, Negative Likert Endorsement Sum Bias	883.55	895.64	0.50	18.58

The best model is (2) Backward Stepwise Regression with AIC as the criterion as the model has the lowest MSE value of 16.56 and highest R^2^ of 0.56 (AIC = 880.06, BIC = 913.33). Although both the AIC and BIC value for this model is not the smallest, it is comparable to the BIC values of the other models. The selected predictors for (2) were Negative Matrix Endorsement Bias and Negative Likert Endorsement Bias. Notably, Negative Matrix Endorsement Bias was also included in the (1) Forward Stepwise Regression with AIC as criterion final model and (3) Forward Stepwise Regression with BIC as criterion final model. Taken together, these findings suggest that having a larger number of words may serve as a more robust predictor for depressive symptoms. The implications of these results will be further discussed in the Discussion section.

### Comparison of different word lists

Regression analysis was conducted to assess the predictive validity of endorsement bias across four different word lists: the 23 overlapping words list, 40 words list from LeMoult’s study, the 60 words list used in Dataset A and the 200 words list used in the matrix for Dataset A. **Table 4** presents the effect of different word lists on endorsement bias measures in Dataset A.

**Table 4:** Effect of word selection on Endorsement Bias measures in Dataset A.

Variable	*B*	*SE*	95% CI		*t*	*p*	Model			
			LL	UL			*R^2^*	*MSE*	*F (df)*	*p*
Negative Endorsement Bias										
23 words list	15.15	1.57	12.06	18.24	9.67	7.18E^-18^	0.379	23.31	25.78 (5,169)	9.35E^-19^
LeMoult word list (40 words)	16.64	1.58	13.52	19.76	10.53	2.89E^-20^	0.418	21.85	30.32 (5,169)	4.42E^-21^
60 words list	15.71	1.45	12.84	18.58	10.78	6.70E^-21^	0.447	20.76	19.17 (8, 166)	5.77E^-22^
200 words list	15.83	1.23	13.40	18.27	12.85	1.11E^-26^	0.528	17.70	26.58 (8, 166)	7.51E^-28^
Positive Endorsement Bias										
23 words list	-15.20	1.58	-18.33	-12.08	-9.59	1.14E^-18^	0.376	23.44	25.41 (5,169)	1.56E^-18^
LeMoult word list	-16.64	1.58	-19.76	-13.52	-10.53	2.89E^-20^	0.418	21.85	30.32 (5,169)	4.42E^-21^
60 words list	-2.29	0.79	-3.86	-0.74	-2.90	4.16E^-3^	0.105	33.60	2.78 (8, 166)	8.76E^-3^
200 words list	15.83	1.23	-18.27	-13.40	-12.85	1.11E^-26^	0.528	17.70	26.58 (8, 166)	7.51E^-28^
Difference in Endorsement Bias										
23 words list	7.59	0.788	6.04	9.15	9.63	8.81E^-18^	0.377	23.37	25.78 (5,169)	1.18E^-18^
LeMoult word list	8.32	0.79	6.76	9.88	10.53	2.89E^-20^	0.418	21.85	20.32 (5,169)	4.42E^-21^
60 words list	3.38	0.60	2.20	4.58	5.62	7.61E^-8^	0.210	29.65	6.30 (8, 166)	1.13E^-7^
200 words list	7.92	0.62	6.70	9.13	12.85	1.11E^-26^	0.528	17.70	26.58 (8, 166)	7.51E^-28^

This table presents the R^2^ values compared across the different word lists.

The results showed that the MSE value for the 200 words list was the lowest among all the word lists analysed across various measures of Endorsement Bias. Specifically, the 200 words list yielded an MSE value of 17.70 (*F* (8, 166) = 26.58, *p* <.001, R^2^ = 0.528) for Negative Endorsement Bias while the MSE values for the 23 words, 40 words and 60 words were 23.31 (*F* (5, 169) = 15.78, *p* <.001, R^2^ = 0.379), 21.85 (*F* (5, 169) = 30.32, *p* <.001, R^2^ = 0.418) and 19.17 (*F* (8, 166) = 20.76, *p* <.001, R^2^ = 0.447), respectively. For Positive Endorsement Bias and Difference in Endorsement Bias measures, a similar trend was observed as the MSE value of the 200 words list was the lowest compared to the other word lists. For the Positive Endorsement Bias model, the MSE value was 23.44 (*F* (5,169) = 25.41, *p* <.001, R^2^ = 0.376) in the 23-word list, 21.85 (*F* (5,169) = 30.32, *p* <.001, R^2^ = 0.418) in the 40-word list, 33.60 (*F* (8, 166) = 2.78, *p* <.001, R^2^ = 0.105) in the 60-word list and 17.70 (*F* (8, 166) = 26.58, *p* <.001, R^2^ = 0.528) in the 200-word list. Similarly, for the Difference in Endorsement Bias model, the rank order of MSE values mirrored that of the Positive Endorsement Bias model, with MSE values of 23.37 (*F* (5,169) = 25.78, *p* <.001, R^2^ = 0.377), 21.85 (*F* (5,169) = 30.32, *p* <.001, R^2^ = 0.418), 29.65 (*F* (8, 166) = 6.30, *p* <.001, R^2^ = 0.210) and 17.70 (*F* (8, 166) = 26.58, *p* <.001, R^2^ = 0.528) for the 23-word, 40-word, 60-word and 200-word list respectively.

## Discussion

The SRET offers multiple measures for assessing SRP and its associations with depressive symptoms. In this study, we investigated several key measures derived from the SRET and its task variations. These measures included endorsement bias measures, RT measures, drift rates, recall measures, recognition measures, as well as matrix measures and Likert measures. The aim was to determine which of these measures had the highest predictive accuracy in predicting depressive symptoms across different samples.

The comparison of SRET measures across the three distinct datasets revealed a trend: while SRET measures demonstrated significant predictive utility within the clinical and healthy controls population (Dataset A), this association was not observed in the university populations (Datasets B and C). An F-test was then conducted to compare the severity of depressive symptoms between the clinical population and the university population. The results revealed a significant difference in depressive symptom severity, indicating that the clinical population exhibited greater depressive symptom severity compared to the university population. This finding suggests that the SRET may hold greater predictive value within populations already experiencing more severe depressive symptoms, rather than in non-clinical samples. This is corroborated by previous research (14, 31) which found that individuals with major depressive disorder (MDD) exhibit greater negative SRP bias, possessing more negative self-schemas compared to non-depressed individuals. Consequently, subsequent analyses were conducted on Dataset A alone as it demonstrated more robust associations with depressive symptoms. Future studies could explore the efficacy of the SRET in non-clinical samples with larger sample sizes, to further validate the use of the SRET beyond clinical populations.

Within Dataset A, the comparative analysis revealed that endorsement bias emerged as one of the strongest SRET measures in predicting depressive symptoms, ranking seventh overall. Notably, it performed better than the proportion of negative and positive words endorsed. This is corroborated by Dainer-Best et al. (12), whose research indicated that endorsement measures were among the strongest predictors of depressive symptoms. Given the multitude of calculation methods for endorsement data, this finding suggests that utilising endorsement bias may enhance the predictive power of endorsement measures in assessing depressive symptoms.

Although conventional RT bias measures significantly predicted depressive symptoms, our findings suggest that drift rates may be better predictors of depressive symptoms. Previous research indicates that depressed individuals often show a heightened attentional bias towards negative stimuli, which can be hypothesised to manifest as quicker responses to negative words due to increased salience for depressed individuals (32). This is supported by Fritzsche et al. (17) and Collins and Winer (14), who revealed that depressed individuals consistently exhibit slower reaction times than their non-depressed counterparts, indicating a prolonged decision-making process when processing self-referential adjectives. These findings complicate the use of RT as a reliable marker for depressive symptoms and underscores the complexity of assessing cognitive processes in MDD. The drift-diffusion model emerged as a better predictor than conventional SRET measures, such as RT bias, ranking at nineteenth place overall. Furthermore, the drift rates also offered reductions in MSE when incrementally added to the base regression model beyond endorsement measures. Consistent with findings from previous research (12), our study did not find robust associations between recall measures and depressive symptoms. This suggests that recall performance may not be a reliable predictor of depressive symptoms across different populations and methodologies. Further investigations are warranted to better understand the role of recall measures in assessing depressive symptomatology.

In our current study, we expanded the conventional SRET task by incorporating additional components such as a recognition task, presenting the SRET in a matrix format and including a Likert scale for a more nuanced analysis of SRP.

For the recognition task, the use of SDT to analyse participants’ recognition of self-descriptive adjectives unexpectedly did not yield better results as compared to calculating the proportion of correctly recognised and endorsed negative and positive words. Negative Recognition Bias has the lowest MSE value out of the recognition bias measures and was ranked 12^th^ overall. ​​It was calculated as the proportion of negative words that were both correctly recognised and endorsed, divided by the total number of negative words correctly recognised as being presented in the previous list. This metric takes into account both recognition accuracy and endorsement tendencies, which have shown to be key for understanding cognitive biases related to depressive symptom severity.

On the other hand, response bias (*c+* or *c-*) quantifies the extent to which a “yes, the word was presented in the previous list” versus “no, the word was not presented” is more probable for positive or negative words endorsed independent of sensitivity. Hence, response bias measures provide insight into participant’s general decision-making tendencies but does not directly account for accuracy or interaction between recognition and endorsement. Therefore, Negative Recognition Bias, which integrate accuracy and endorsement measures more directly might have performed better at capturing the maladaptive negative self-schemas.

The present findings suggest that the Negative Matrix Endorsement Bias and the Difference in Likert Endorsement Sum bias variables are the most robust SRET measures in terms of their predictive accuracy for depressive symptoms as they have the lowest MSE values and highest R^2^ values, ranking the top among other SRET measures. Furthermore, when these measures were incrementally added to the base regression model, the MSE consistently decreased, and the R^2^ value increased, indicating a significant improvement in the model’s explanatory power to depressive symptoms.

The study employed forward and backward stepwise regression models to determine the best subset of measures. Interestingly, Negative Matrix Endorsement Bias consistently appeared in three of the four models, regardless of whether AIC or BIC was used as a stopping criterion, suggesting its robust predictive power across different model selection techniques. Therefore, Negative Matrix Endorsement Bias, which is indicative of a depressotypic processing style (33), might be a reliable marker for screening for depressive symptoms. Consistent with our findings, the Endorsement Bias measures derived from the 200 words list demonstrated superior predictive validity, as evidenced by having the lowest MSE value compared to the other words lists.

These results have several implications to the SRET. First, expanding the word list in the SRET appears to be more beneficial for predicting depressive symptoms. Existing literature has well-documented that depressogenic patterns of self-schemas are associated with concurrent and prospective depressive symptoms in clinical samples of depressed adults (16, 17), adolescents (34), as well as in non-clinical samples of youth (20, 35). By expanding the word list, our study may have captured a broader spectrum of depressogenic self-referential representations, allowing for a richer representation of individuals’ self-schemas, potentially enhancing the sensitivity of the SRET for screening for depressive symptoms (7). On average, each of the task components lasted less than 5 minutes regardless of the length of the word list, suggesting that the 200-word list did substantially increase respondent burden. Furthermore, the expanded word list could help in identifying subtle nuances in self-referential processing that might be missed with a shorter list. Future research should explore the optimal length and content of word lists in SRET to maximise its predictive power. Clinically, using a more comprehensive word list in SRET could improve the accuracy of depressive symptom screening and better inform treatment strategies. Next, the presentation of the SRET stimuli in a matrix format might have facilitated the integration of self-referential information as the adjectives were shown simultaneously. A study by Bharti et al. (36) found that simultaneous presentation affects the organisation of stimuli in the visual working memory differently than sequential presentation and these cognitive processes of memory are closely related to self-referential processing (37, 38). Hence, the matrix format may have allowed for more effective encoding and retrieval of self-related information. Although there are existing studies that have investigated the neural activity associated with SRET (39), future research could explore the neural underpinnings associated with sequential versus simultaneous presentation of the self–descriptive words to better understand these cognitive processes and their impact on self-referential processing.

Utilising a Likert-response format for the SRET demonstrated better predictive validity compared to the traditional binary-response format of the SRET. The Difference in Likert Endorsement Sum Bias variable performed the best among the various SRET measures. While previous research suggests that a binary response format often imposes the least response burden on participants (40), in our study, we found that the Likert-response options allows for more nuanced self-referential judgements. A study conducted with older adults endorsing depressive symptoms tested both Likert response options and binary response options and the authors concluded that binary response options may have increased response burden and cognitive load on their participants as the individual must decide if their experience was sufficient enough to warrant a complete endorsement of the symptom (41). Similarly, in the context of the SRET, binary responses may fail to fully capture the subtleties of the participants’ self-schemas. In our study, administering Likert response options after participants selected words in the matrix may have provided the participants the opportunity to clarify the degree of endorsement, capturing the complexity of the participants’ self-schemas more effectively than binary options. This is supported by the current results where using the full range of Likert scores, rather than collapsing the responses into binary categories produced the best predictive measure, with the Difference in Likert Endorsement Sum Bias emerging as the best model for predicting depressive symptom categories.

Furthermore, as seen in **Figure 1**, we hypothesise that the differences arising between Likert response categories and binary-coded response categories may be associated with depression. Notably, these differences do not appear to have been adequately captured by novel measures such as SDT or drift rates derived from reaction time (RT) data. The findings suggest that a Likert response format may offer superior sensitivity compared to a binary response format, not only in this study but potentially in other similar tasks. Future research could further explore the utility of Likert-response formats in the SRET and examine whether they confer additional benefits as compared to the traditional binary-response options in capturing SRP.

## Conclusion

In summary, our study provides a head-to-head comparison of the predictive accuracy and incremental predictive value between SRET measures in the prediction of depressive symptoms. We find that an expanded word list and a Likert response format rather than binary response format improved the sensitivity of the SRET for depressive symptoms. Self-descriptive judgements without recall or memory measures may be sufficient for prediction of depressive symptoms. The implications of this study are significant for both research and the development of translational applications of the SRET. By refining the SRET with innovative measures and formats, we can improve its efficiency as a potential assessment in depression. Future research should continue to explore the neural and cognitive mechanisms underlying different presentation formats and further optimise the SRET by refining word selection to maximise its predictive accuracy. In conclusion, our study contributes to a deeper understanding of cognitive biases in depression and demonstrates the value of our adaptations to the SRET.

## Data Availability

The raw data supporting the conclusions of this article will be made available by the authors, without undue reservation.
