# Supplementary material for: Evaluating the relative predictive validity of measures of self-referential processing for depressive symptom severity
Source: Front Psychiatry. 2025 Feb 10;15:1463116. doi: 10.3389/fpsyt.2024.1463116 (PMC11847881; doi:10.3389/fpsyt.2024.1463116)
Supplement: Supplementary file 1 [file Table1.docx]

***Supplementary Material***

**Demographics**

The demographic characteristics of participants of each dataset and a breakdown of patient participants' medication status, including counts for specific types of medications are presented in Supplementary Table 1. Significant differences were observed in age distribution among the datasets. Specifically, Dataset A had a significantly different age distribution compared to Dataset B (*t*(233) = 7.00, 95%CI [6.50, 11.58], *p* < .001), and also when compared to Dataset C (*t*(269) = 7.77 , 95%CI [6.01, 10.08], *p* < .001). The age distribution between Dataset B and Dataset C was also significantly different (*t*(156) = -2.615 , 95%CI [-1.75, -0.24], *p* < .05).

A chi-square test was conducted to examine the association between gender and group membership across the three datasets. The analysis revealed a statistically significant association between gender and group membership (*χ*2 = 11.95, df = 2, *p* < .05). Chi-square test was also conducted to assess the association between race and group membership across the combined dataset. The analysis revealed a non-significant association between race and datasets (*χ*2 = 11.54, df = 6, *p* = .073). However, upon closer examination of pairwise comparisons, a statistically significant difference was found between Dataset A and Datasets B and C combined (*χ*2 = 10.55, df = 3, *p* < .05. This suggests that there may be variations in the racial distribution between individuals in the clinical setting compared to those in the university setting. Therefore, the effect of age, gender and race distribution was checked and controlled in subsequent analyses.

**[Supplementary Table 1]**

**SUPPLEMENTARY TABLE 1 |** Demographics of participants across datasets

|  |  |  |  |  |  |  |  |
| --- | --- | --- | --- | --- | --- | --- | --- |
| Dataset | A (*N* = 191) | | B (*N* = 61) | | C (*N* = 97) | |  |
|  | *n* | *%* | *n* | *%* | *n* | *%* |  |
| Gender |  |  |  |  |  |  |  |
| Male | 89 | 47.3% | 21 | 34.4% | 30 | 30.9% |  |
| Female | 86 | 45.7% | 40 | 65.6% | 67 | 69.1% |  |
| Missing | 13 | 6.9% | - | - | - | - |  |
|  |  |  |  |  |  |  |  |
| Ethnicity |  |  |  |  |  |  |  |
| Chinese | 141 | 75.0% | 57 | 93.4% | 85 | 87.6% |  |
| Malay | 16 | 8.5% | - | - | 2 | 2.06% |  |
| Indian | 9 | 4.8% | 2 | 3.27% | 5 | 5.15% |  |
| Others | 9 | 4.8% | 2 | 3.27% | 5 | 5.15% |  |
| Missing | 13 | 6.9% | - | - | - | - |  |
|  |  |  |  |  |  |  |  |
|  | *M* | *SD* | *M* | *SD* | *M* | *SD* |  |
| Age | 31.50 | 10.00 | 22.45 | 1.89 | 23.45 | 2.56 |  |
|  |  | |  | |  | |  |
| Studies | CholDep  (N = 52) | | XChange  (N = 84) | | PRE-EMPT  (N = 52) | |  |
| Medication Count | *n* | | *n* | | *n* | |  |
| SSRI | 22 | | 40 | | 14 | |  |
| SNRI | 4 | | 7 | | 1 | |  |
| SARI/SMS/MaSSA/NaSSI/NDRI | 11 | | 13 | | 4 | |  |
| TCA | 2 | | 5 | | 1 | |  |
| Benzodiazepines | 5 | | 14 | | 3 | |  |
| Non-Benzodiazepine Hypnotic | 0 | | 3 | | 1 | |  |
| Anticonvulsant | 9 | | 7 | | 0 | |  |
| Atypical Antipsychotic | 4 | | 12 | | 4 | |  |
| Typical Antipsychotic | 1 | | 3 | | 0 | |  |
| Beta Blocker | 3 | | 1 | | 1 | |  |
| Antihistamines | 2 | | 1 | | 3 | |  |
| Stimulants | 1 | | 2 | | 1 | |  |
| No Medications/Incomplete Information | 30 | | 22 | | 26 | |  |
|  |  | |  | |  | |  |
| Studies | CholDep  (N = 52) | | XChange  (N = 84) | | PRE-EMPT  (N = 52) | |  |
| Diagnosis count | *n* | | *n* | | *n* | |  |
| MDD | 10 | | 13 | | 16 | |  |
| Anxiety | 7 | | 4 | | 6 | |  |
| Persistent Depressive Disorder | 2 | | 2 | | 1 | |  |
| Bipolar Disorder | 1 | | 1 | | 0 | |  |
| OCD | 1 | | 2 | | 0 | |  |
| ADHD | 0 | | 0 | | 1 | |  |
| PTSD/Panic Attack/Agoraphobia/Panic Disorder | 1 | | 2 | | 3 | |  |
| BPD/Borderline Traits | 1 | | 1 | | 2 | |  |
| Insomnia | 1 | | 0 | | 0 | |  |
| Eating Disorder | 0 | | 1 | | 0 | |  |
| Gender Dysphoria | 0 | | 1 | | 0 | |  |
| No Diagnosis/Incomplete Information | 36 | | 67 | | 33 | |  |
|  |  | |  | |  | |  |

*Note.* Dataset A consisted of three distinct research studies: CholDep, Xchange, and PRE-EMPT.
