# Supplementary material for: Evaluating the relative predictive validity of measures of self-referential processing for depressive symptom severity
Source: Front Psychiatry. 2025 Feb 10;15:1463116. doi: 10.3389/fpsyt.2024.1463116 (PMC11847881; doi:10.3389/fpsyt.2024.1463116)
Supplement: Supplementary file 2 [file Table2.docx]

***Supplementary Material***

**[Supplementary Table 2]**

**SUPPLEMENTARY TABLE 2 |** Regression Analysis of Endorsement Bias for Full Word List with Depressive Symptoms

|  |  |  |  |  |  |  |  |  |  |  |
| --- | --- | --- | --- | --- | --- | --- | --- | --- | --- | --- |
|  |  |  | 95% CI | |  |  | Model | | | |
| Variable | *B* | *SE* | LL | UL | *t* | *p* | *R^2^* | MSE | *F (df)* | *p* |
| Positive Endorsement Bias |  |  |  |  |  |  |  |  |  |  |
| Dataset A | -2.29 | 0.79 | -3.86 | -0.74 | -2.90 | 4.16E^-3^*** | 0.105 | 33.60 | 2.78 (8, 166) | 8.76E^-3^*** |
| Dataset B | -1.35 | 2.01 | -5.40 | 2.69 | -0.67 | .51 | 0.176 | 15.18 | 2.62 (5, 49) | .352 |
| Dataset C | -5.17 | 3.67 | -12.45 | 2.11 | -1.41 | .162 | 0.063 | 21.39 | 1.00 (7, 90) | .162 |
| Negative Endorsement Bias |  |  |  |  |  |  |  |  |  |  |
| Dataset A | 15.71 | 1.45 | 12.84 | 18.58 | 10.78 | 6.70E^-21^*** | 0.447 | 20.76 | 19.17 (8, 166) | 5.77E^-22^*** |
| Dataset B | 1.35 | 2.01 | -2.69 | 5.40 | 0.67 | .51 | 0.176 | 15.18 | 2.62 (5, 49) | .352 |
| Dataset C | 5.17 | 3.67 | -2.11 | 12.45 | 1.41 | .16 | 0.063 | 21.39 | 1.00 (7, 90) | .162 |
| Difference in negative endorsement bias and positive endorsement bias |  |  |  |  |  |  |  |  |  |  |
| Dataset A | 3.38 | 0.60 | 2.20 | 4.58 | 5.62 | 7.61E^-8^ | 0.210 | 29.65 | 6.30 (8, 166) | 1.13E^-7^ *** |
| Dataset B | 0.62 | 1.02 | -1.43 | 2.67 | 0.61 | .55 | 0.128 | 15.87 | 2.05 (5, 56) | .369 |
| Dataset C | 2.58 | 1.83 | -1.06 | 6.22 | 1.41 | .16 | 0.063 | 21.39 | 1.00 (7, 90) | .166 |
|  |  |  |  |  |  |  |  |  |  |  |

Note. *** *p <* .001.
