# Supplementary material for: Evaluating the relative predictive validity of measures of self-referential processing for depressive symptom severity
Source: Front Psychiatry. 2025 Feb 10;15:1463116. doi: 10.3389/fpsyt.2024.1463116 (PMC11847881; doi:10.3389/fpsyt.2024.1463116)
Supplement: Supplementary file 3 [file Table3.docx]

***Supplementary Material***

**[Supplementary Table 3]**

**SUPPLEMENTARY TABLE 3 |** Regression Analysis of RT Bias for Full Word List with Depressive Symptoms

|  |  |  |  |  |  |  |  |  |  |  |
| --- | --- | --- | --- | --- | --- | --- | --- | --- | --- | --- |
|  |  |  | 95% CI | |  |  | Model | | | |
| Variable | *B* | *SE* | LL | UL | *t* | *p* | *R^2^* | MSE | *F (df)* | *p* |
| Positive RT Bias |  |  |  |  |  |  |  |  |  |  |
| Dataset A | -6545.39 | 1138.08 | -8792.37 | -4298.41 | -5.75 | 4.16E^-8^*** | 0.216 | 29.44 | 6.52  (8, 166) | 5.49E^-8^ *** |
| Dataset B | 0.65 | 0.98 | -1.31 | 2.61 | 0.67 | .508 | 0.129 | 15.84 | 2.07 (5, 56) | .981 |
| Dataset C | 802.16 | 2517.92 | -4200.13 | 5804.45 | 0.32 | .751 | 0.043 | 21.84 | 0.68 (7, 90) | .717 |
| Negative RT Bias |  |  |  |  |  |  |  |  |  |  |
| Dataset A | 5497.89 | 953.00 | 3616.32 | 7379.46 | 5.76 | 3.81E^-8^*** | 0.216 | 29.41 | 6.55  (8, 166) | 3.80E^-9^*** |
| Dataset B | 0.26 | 1.07 | -1.90 | 2.41 | 0.24 | .813 | 0.123 | 15.95 | 1.96 (5, 56) | .957 |
| Dataset C | 467.57 | 2043.61 | -3592.41 | 4527.55 | 0.23 | .820 | 0.043 | 21.85 | 0.67 (7, 90) | .845 |
|  |  |  |  |  |  |  |  |  |  |  |

Note. *** *p <* .001.
