# Supplementary material for: Evaluating the relative predictive validity of measures of self-referential processing for depressive symptom severity
Source: Front Psychiatry. 2025 Feb 10;15:1463116. doi: 10.3389/fpsyt.2024.1463116 (PMC11847881; doi:10.3389/fpsyt.2024.1463116)
Supplement: Supplementary file 4 [file Table4.docx]

***Supplementary Material***

**[Supplementary Table 4]**

**SUPPLEMENTARY TABLE 4 |** Regression Analysis of Recall Bias for Full Word List with Depressive Symptoms

|  |  |  |  |  |  |  |  |  |  |  |
| --- | --- | --- | --- | --- | --- | --- | --- | --- | --- | --- |
|  |  |  | 95% CI | |  |  | Model | | | |
| Variable | *B* | *SE* | LL | UL | *t* | *p* | *R^2^* | MSE | *F (df)* | *p* |
| Proportion of Negative Endorsed and Recalled Words to Total Endorsed Words |  |  |  |  |  |  |  |  |  |  |
| Dataset A | 18.96 | 4.41 | 10.26 | 27.67 | 4.30 | 4.58E^-5^*** | 0.154 | 31.77 | 4.30 (8, 166) | 4.58E^-5^*** |
| Dataset C | 11.38 | 7.60 | -3.72 | 26.47 | 1.50 | .14 | 0.065 | 21.33 | 1.05 (7, 90) | .124 |
| Proportion of Positive Endorsed and Recalled Words to Total Endorsed Words |  |  |  |  |  |  |  |  |  |  |
| Dataset A | -10.00 | 3.72 | -17.34 | -2.66 | -2.691 | 7.68E^-3^*** | 0.099 | 33.83 | 2.59 (8, 166) | 2.52E^-3^*** |
| Dataset C | 6.72 | 5.39 | -3.98 | 17.43 | 1.25 | 0.22 | 0.058 | 21.49 | 0.93 (7, 90) | .279 |
| Negative Recall Bias |  |  |  |  |  |  |  |  |  |  |
| Dataset A | 8.57 | 1.37 | 5.87 | 11.29 | 6.25 | 4.09E^-9^*** | 0.241 | 28.39 | 6.72 (8, 148) | 1.03E^-9^*** |
| Dataset C | 1.12 | 1.69 | -2.25 | 4.48 | 0.66 | 0.51 | 0.051 | 21.76 | 0.72 (7, 80) | .536 |
|  |  |  |  |  |  |  |  |  |  |  |

Note. *** *p <* .001.
