# Supplementary material for: Evaluating the relative predictive validity of measures of self-referential processing for depressive symptom severity
Source: Front Psychiatry. 2025 Feb 10;15:1463116. doi: 10.3389/fpsyt.2024.1463116 (PMC11847881; doi:10.3389/fpsyt.2024.1463116)
Supplement: Supplementary file 5 [file Table5.docx]

***Supplementary Material***

**[Supplementary Table 5]**

**SUPPLEMENTARY TABLE 5** | Regression Analysis of Endorsement bias for 23 overlapping word list with Depressive Symptoms in all datasets

|  |  |  | 95% CI | |  |  | Model | | | |
| --- | --- | --- | --- | --- | --- | --- | --- | --- | --- | --- |
| Variable | *B* | *SE* | LL | UL | *t* | *p* | *R^2^* | MSE | *F (df)* | *p* |
| Proportion of Negative Words endorsed |  |  |  |  |  |  |  |  |  |  |
| Dataset A (Patients) | 9.55 | 1.74 | 6.10 | 13.00 | 5.48 | 2.22E^-07^ | 0.25 | 21.39 | 10.68  (5, 125) | 1.49E^-08^*** |
| Dataset A (Healthy Controls) | 5.92 | 2.63 | 0.603 | 11.24 | 2.25 | .03 | 0.177 | 10.14 | 2.10  (5, 39) | .033* |
| Dataset B | 0.52 | 2.11 | -3.71 | 4.75 | 0.25 | .806 | 0.137 | 15.70 | 1.75  (6, 55) | .897 |
| Dataset C | 4.15 | 2.14 | -0.107 | 8.40 | 1.94 | -.0566 | 0.080 | 20.99 | 1.31  (5, 92) | .054 |
| Combined (Dataset B + Dataset C) | 2.28 | 1.52 | -0.732 | 5.29 | 1.50 | .137 | 0.046 | 20.32 | 1.04  (8, 150) | .154 |
| Proportion of Positive Words endorsed |  |  |  |  |  |  |  |  |  |  |
| Dataset A (Patients) | -5.41 | 1.65 | -8.68 | -2.14 | -3.27 | 1.38E^-03^ | 0.148 | 24.4 | 5.44  (5, 125) | 1.80E^-03^*** |
| Dataset A (Healthy Controls) | -8.68 | 1.77 | -12.27 | -5.10 | -4.91 | 1.69E^-05^ | 0.425 | 7.09 | 7.20  (5, 39) | 5.54E^-05^*** |
| Dataset B | -0.32 | 1.68 | -3.69 | 4.75 | -0.19 | .849 | 0.137 | 15.70 | 1.74  (6, 55) | .570 |
| Dataset C | -0.505 | 2.02 | -4.53 | 3.52 | -0.25 | .803 | 0.043 | 21.85 | 0.669  (7, 90) | .833 |
| Combined (Dataset B + Dataset C) | -1.038 | 1.24 | -3.50 | 1.42 | -0.834 | .406 | 0.036 | 20.53 | 0.808  (8, 150) | .332 |
| Negative Endorsement Bias |  |  |  |  |  |  |  |  |  |  |
| Dataset A (Patients) | 12.53 | 2.43 | 7.73 | 17.3 | 5.17 | 9.20E^-07^ | 0.24 | 21.87 | 9.76  (5, 125) | 5.05E^-07^*** |
| Dataset A (Healthy Controls) | 7.73 | 1.77 | 4.15 | 11.32 | 4.36 | 9.11E^-05^ | 0.375 | 7.70 | 5.85  (5, 39) | 2.36E^-04^*** |
| Dataset B | 1.32 | 2.15 | -3.00 | 5.63 | 0.61 | .542 | 0.184 | 15.03 | 2.17  (6, 48) | .426 |
| Dataset C | 1.96 | 2.17 | -2.35 | 6.27 | 0.902 | .369 | 0.051 | 21.67 | 0.799  (7, 90) | .276 |
| Combined (Dataset B + Dataset C) | 2.02 | 1.56 | -1.06 | 5.09 | 1.30 | .196 | 0.0391 | 20.69 | 0.83  (8, 143) | .166 |
| Positive Endorsement Bias |  |  |  |  |  |  |  |  |  |  |
| Dataset A (Patients) | -0.49 | 0.10 | -0.69 | -0.29 | -4.85 | 3.49E^-06^ | 0.22 | 22.4 | 8.96  (5, 124) | 4.15E^-06^*** |
| Dataset A (Healthy Controls) | -0.20 | 0.072 | -0.35 | -0.055 | -2.80 | 8.48E^-03^ | 0.29 | 8.43 | 3.39  (5, 33) | .025* |
| Dataset B | -1.32 | 2.15 | -5.63 | 3.00 | -0.61 | .542 | 0.184 | 15.03 | 2.17  (6, 48) | .426 |
| Dataset C | -1.96 | 2.17 | -6.27 | 2.35 | -0.902 | .369 | 0.051 | 21.67 | 0.80  (7, 90) | .276 |
| Combined (Dataset B + Dataset C) | -2.02 | 1.56 | -5.09 | 1.06 | –1.30 | .196 | 0.039 | 20.69 | 0.83  (8, 143) | .166 |
| Difference in Endorsement Bias |  |  |  |  |  |  |  |  |  |  |
| Dataset A (Patients) | 0.48 | 0.098 | 0.29 | 0.68 | 4.90 | 2.91E^-06^ | 0.226 | 22.4 | 9.07  (5, 124) | 3.33E^-06^*** |
| Dataset A (Healthy Controls) | 0.20 | 0.070 | 0.057 | 0.34 | 2.84 | 7.68E^-03^ | 0.30 | 8.38 | 3.46  (5, 33) | .023* |
| Dataset B | 0.66 | 1.07 | -1.50 | 2.82 | 0.61 | .542 | 0.184 | 15.03 | 2.17  (6, 48) | .426 |
| Dataset C | 0.979 | 1.08 | -1.18 | 3.13 | 0.902 | .369 | 0.051 | 21.679 | 0.799  (7, 90) | .276 |
| Combined (Dataset B + Dataset C) | 1.01 | 0.78 | -0.537 | 2.55 | 1.30 | .196 | 0.039 | 20.69 | 0.831  (8,143) | .166 |
|  |  |  |  |  |  |  |  |  |  |  |

Note. * *p <* .05, *** *p <* .001.
