# Supplementary material for: Evaluating the relative predictive validity of measures of self-referential processing for depressive symptom severity
Source: Front Psychiatry. 2025 Feb 10;15:1463116. doi: 10.3389/fpsyt.2024.1463116 (PMC11847881; doi:10.3389/fpsyt.2024.1463116)
Supplement: Supplementary file 6 [file Table6.docx]

***Supplementary Material***

**[Supplementary Table 6]**

**SUPPLEMENTARY TABLE 6** | Regression Analysis of RT Bias for 23 overlapping word list with Depressive Symptoms in all datasets

|  |  |  | 95% CI | |  |  | Model | | | |
| --- | --- | --- | --- | --- | --- | --- | --- | --- | --- | --- |
| Variable | *B* | *SE* | LL | UL | *t* | *p* | *R^2^* | MSE | *F (df)* | *p* |
| Negative RT Bias |  |  |  |  |  |  |  |  |  |  |
| Dataset A (Patients) | -1.04 | 0.70 | -2.42 | 0.34 | -1.50 | .137 | 0.0917 | 26.07 | 3.15  (5,125) | .0794 |
| Dataset A (Healthy Controls) | 1.41 | 1.02 | -0.65 | 3.46 | 1.38 | .175 | 0.113 | 10.92 | 1.25  (5,39) | .158 |
| Dataset B | -1137.18 | 1277.80 | -3709.25 | 1434.90 | -.89 | .378 | 0.201 | 15.26 | 2.31  (6, 46) | .408 |
| Dataset C | -1409.21 | 1150.66 | -3696.27 | 877.85 | -1.22 | .224 | 0.081 | 20.62 | 1.27  (7, 87) | .130 |
| Combined (Dataset B + Dataset C) | -1393.57 | 878.70 | -3131.02 | 343.88 | -1.59 | .115 | 0.070 | 20.03 | 1.49  (8, 138) | .110 |
| Positive RT Bias |  |  |  |  |  |  |  |  |  |  |
| Dataset A (Patients) | 0.366 | 0.65 | -0.928 | 1.66 | 0.560 | .577 | 0.078 | 26.47 | 2.63  (5, 125) | .546 |
| Dataset A (Healthy Controls) | -0.586 | 0.79 | -2.19 | 1.02 | -0.74 | .465 | 0.083 | 11.30 | .881  (5, 39) | .476 |
| Dataset B | 680.54 | 1052.45 | -1446.54 | 2807.63 | 0.65 | .522 | 0.177 | 14.86 | 1.72  (6, 40) | .813 |
| Dataset C | -66.36 | 1096.25 | -2259.19 | 2126.48 | -0.061 | .952 | 0.062 | 21.11 | 0.655  (7, 60) | .672 |
| Combined (Dataset B + Dataset C) | -196.5 | 770.28 | -1723.84 | 1330.81 | -.255 | .799 | 0.021 | 20.38 | 0.321 (8, 105) | .809 |
|  |  |  |  |  |  |  |  |  |  |  |
