# Supplementary material for: Evaluating the relative predictive validity of measures of self-referential processing for depressive symptom severity
Source: Front Psychiatry. 2025 Feb 10;15:1463116. doi: 10.3389/fpsyt.2024.1463116 (PMC11847881; doi:10.3389/fpsyt.2024.1463116)
Supplement: Supplementary file 7 [file Table7.docx]

***Supplementary Material***

**[Supplementary Table 7]**

**SUPPLEMENTARY TABLE 7** | Regression Analysis of Endorsement bias and RT bias for 23 overlapping word list with Depressive Symptoms in Dataset A only

|  |  |  |  |  |  |  |  |  |  |  |
| --- | --- | --- | --- | --- | --- | --- | --- | --- | --- | --- |
|  |  |  | 95% CI | |  |  | Model | | | |
| Variable | *B* | *SE* | LL | UL | *t* | *p* | *R^2^* | MSE | *F (df)* | *p* |
| Proportion of Negative Words Endorsed | 13.59 | 10.02 | 10.91 | 16.27 | -7.09 | 9.04E^-191^ | 0.414 | 22.01 | 16.73  (8, 166) | 4.90E^-20^*** |
| Proportion of Positive Words Endorsed | -9.911 | 1.40 | -12.67 | -7.15 | -7.09 | 3.61E^-11^ | 0.278 | 27.10 | 9.13  (8, 166) | 9.11E^-12^*** |
| Negative Endorsement Bias | 15.17 | 1.58 | 12.05 | 18.30 | 9.58 | 1.36E^-17^ | 0.394 | 22.73 | 15.44  (8, 166) | 9.36E^-19^*** |
| Positive Endorsement Bias | -0.51 | 0.070 | -0.64 | -0.37 | -7.22 | 2.01E^-11^ | 0.287 | 25.62 | 9.16  (8, 159) | 3.87E^-12^*** |
| Difference in Endorsement Bias | 0.50 | 0.068 | 0.362 | 0.629 | 7.31 | 1.23E^-11^ | 0.292 | 25.46 | 9.36  (8, 159) | 2.31E^-12^*** |
| Negative RT bias | -1.34 | 0.75 | -2.81 | -0.13 | -1.80 | .074 | 0.077 | 34.64 | 1.98  (8, 166) | .093 |
| Positive RT bias | 0.26 | 0.66 | -1.04 | 1.57 | 0.40 | .69 | 0.06 | 35.28 | 1.52  (8, 166) | .73 |
|  |  |  |  |  |  |  |  |  |  |  |

Note. *** *p <* .001.
