# Supplementary material for: Evaluating the relative predictive validity of measures of self-referential processing for depressive symptom severity
Source: Front Psychiatry. 2025 Feb 10;15:1463116. doi: 10.3389/fpsyt.2024.1463116 (PMC11847881; doi:10.3389/fpsyt.2024.1463116)
Supplement: Supplementary file 8 [file Table8.docx]

**SUPPLEMENTARY TABLE 8 |** Pearson's Correlation Coefficients between Age, QIDS-16-SR scores and SRET metrics

| Variable | 1 | 2 | 3 | 4 | 5 | 6 | 7 | 8 | 9 | 10 | 11 | 12 | 13 | 14 | 15 | 16 | 17 | 18 | 19 | 20 | 21 | 22 | 23 | 24 | 25 | 26 | 27 | 28 | 29 | 30 | 31 | 32 |
| --- | --- | --- | --- | --- | --- | --- | --- | --- | --- | --- | --- | --- | --- | --- | --- | --- | --- | --- | --- | --- | --- | --- | --- | --- | --- | --- | --- | --- | --- | --- | --- | --- |
| 1. Age | - |  |  |  |  |  |  |  |  |  |  |  |  |  |  |  |  |  |  |  |  |  |  |  |  |  |  |  |  |  |  |  |
| 2. QIDS-16-SR | -0.01 | - |  |  |  |  |  |  |  |  |  |  |  |  |  |  |  |  |  |  |  |  |  |  |  |  |  |  |  |  |  |  |
| 3. Neg-EndRec/Total-End | -0.17* | 0.27*** | - |  |  |  |  |  |  |  |  |  |  |  |  |  |  |  |  |  |  |  |  |  |  |  |  |  |  |  |  |  |
| 4. Pos-EndRec/Total-End | -0.16* | -0.25*** | 0.59*** | - |  |  |  |  |  |  |  |  |  |  |  |  |  |  |  |  |  |  |  |  |  |  |  |  |  |  |  |  |
| 5. Negative Recall Bias | -0.08 | 0.46*** | 0.33*** | -0.44*** | - |  |  |  |  |  |  |  |  |  |  |  |  |  |  |  |  |  |  |  |  |  |  |  |  |  |  |  |
| 6. Negative Endorsement Bias | 0 | 0.65*** | 0.27*** | -0.33*** | 0.71*** | - |  |  |  |  |  |  |  |  |  |  |  |  |  |  |  |  |  |  |  |  |  |  |  |  |  |  |
| 7. Positive Endorsement Bias | 0.08 | -0.2** | -0.12 | 0.09 | -0.36*** | -0.36*** | - |  |  |  |  |  |  |  |  |  |  |  |  |  |  |  |  |  |  |  |  |  |  |  |  |  |
| 8. Neg-End/Total-Neg | -0.01 | 0.63*** | 0.26*** | -0.28*** | 0.58*** | 0.88*** | -0.04 | - |  |  |  |  |  |  |  |  |  |  |  |  |  |  |  |  |  |  |  |  |  |  |  |  |
| 9. Pos-End/Total-Pos | -0.02 | -0.54*** | -0.2** | 0.29*** | -0.64*** | -0.83*** | 0.68*** | -0.55*** | - |  |  |  |  |  |  |  |  |  |  |  |  |  |  |  |  |  |  |  |  |  |  |  |
| 10. *c-* | 0.08 | -0.46*** | -0.3*** | 0.39*** | -0.45*** | -0.62*** | -0.02 | -0.66*** | 0.37*** | - |  |  |  |  |  |  |  |  |  |  |  |  |  |  |  |  |  |  |  |  |  |  |
| 11. *c+* | -0.01 | 0.41*** | 0.3*** | -0.34*** | 0.53*** | 0.63*** | -0.43*** | 0.44*** | -0.68*** | -0.18* | - |  |  |  |  |  |  |  |  |  |  |  |  |  |  |  |  |  |  |  |  |  |
| 12. *c+* minus *c-* | -0.06 | 0.57*** | 0.39*** | -0.47*** | 0.64*** | 0.81*** | -0.27*** | 0.72*** | -0.68*** | -0.77*** | 0.77*** | - |  |  |  |  |  |  |  |  |  |  |  |  |  |  |  |  |  |  |  |  |
| 13. Negative Recognition Bias | -0.04 | 0.6*** | 0.22** | -0.27*** | 0.56*** | 0.78*** | -0.14 | 0.82*** | -0.56*** | -0.63*** | 0.5*** | 0.73*** | - |  |  |  |  |  |  |  |  |  |  |  |  |  |  |  |  |  |  |  |
| 14. Positive Recognition Bias | 0.03 | -0.5*** | -0.18* | 0.31*** | -0.61*** | -0.75*** | 0.49*** | -0.58*** | 0.81*** | 0.48*** | -0.68*** | -0.75*** | -0.46*** | - |  |  |  |  |  |  |  |  |  |  |  |  |  |  |  |  |  |  |
| 15. *v-* | 0.11 | 0.45*** | 0.22** | -0.46*** | 0.45*** | 0.74*** | -0.08 | 0.8*** | -0.5*** | -0.54*** | 0.4*** | 0.61*** | 0.65*** | -0.51*** | - |  |  |  |  |  |  |  |  |  |  |  |  |  |  |  |  |  |
| 16. *v+* | 0.04 | -0.47*** | -0.27*** | 0.37*** | -0.51*** | -0.69*** | 0.5*** | -0.48*** | 0.8*** | 0.41*** | -0.54*** | -0.62*** | -0.49*** | 0.7*** | -0.27*** | - |  |  |  |  |  |  |  |  |  |  |  |  |  |  |  |  |
| 17. Negative RT Bias | -0.05 | 0.44*** | 0.21** | -0.16* | 0.46*** | 0.66*** | 0.02 | 0.79*** | -0.36*** | -0.51*** | 0.32*** | 0.55*** | 0.65*** | -0.38*** | 0.64*** | -0.38*** | - |  |  |  |  |  |  |  |  |  |  |  |  |  |  |  |
| 18. Positive RT Bias | -0.05 | -0.4*** | -0.16* | 0.22** | -0.57*** | -0.63*** | 0.58*** | -0.38*** | 0.81*** | 0.31*** | -0.51*** | -0.53*** | -0.4*** | 0.67*** | -0.45*** | 0.66*** | -0.05 | - |  |  |  |  |  |  |  |  |  |  |  |  |  |  |
| 19. Negative Likert Endorsement Bias | 0 | 0.62*** | 0.22** | -0.21** | 0.43*** | 0.7*** | -0.05 | 0.75*** | -0.49*** | -0.52*** | 0.43*** | 0.62*** | 0.7*** | -0.48*** | 0.56*** | -0.44*** | 0.58*** | -0.39*** | - |  |  |  |  |  |  |  |  |  |  |  |  |  |
| 20. Positive Likert Endorsement Bias | 0.05 | -0.56*** | -0.12 | 0.32*** | -0.56*** | -0.72*** | 0.47*** | -0.58*** | 0.79*** | 0.47*** | -0.57*** | -0.68*** | -0.53*** | 0.73*** | -0.5*** | 0.64*** | -0.37*** | 0.6*** | -0.41*** | - |  |  |  |  |  |  |  |  |  |  |  |  |
| 21. Difference in Likert Endorsement Bias | -0.03 | 0.69*** | 0.2** | -0.32*** | 0.59*** | 0.85*** | -0.33*** | 0.78*** | -0.77*** | -0.58*** | 0.6*** | 0.76*** | 0.73*** | -0.73*** | 0.62*** | -0.64*** | 0.55*** | -0.6*** | 0.81*** | -0.87*** | - |  |  |  |  |  |  |  |  |  |  |  |
| 22. Neg-Likert-End/ Total-Neg | -0.04 | 0.7*** | 0.18* | -0.35*** | 0.62*** | 0.86*** | -0.31*** | 0.78*** | -0.74*** | -0.6*** | 0.59*** | 0.77*** | 0.74*** | -0.7*** | 0.64*** | -0.61*** | 0.53*** | -0.61*** | 0.78*** | -0.8*** | 0.94*** | - |  |  |  |  |  |  |  |  |  |  |
| 23. Negative Matrix Endorsement Bias | -0.04 | 0.7*** | 0.21** | -0.34*** | 0.65*** | 0.88*** | -0.32*** | 0.79*** | -0.76*** | -0.62*** | 0.61*** | 0.8*** | 0.75*** | -0.72*** | 0.67*** | -0.62*** | 0.54*** | -0.62*** | 0.76*** | -0.81*** | 0.93*** | 0.98*** | - |  |  |  |  |  |  |  |  |  |
| 24. Neg-Matrix-End/ Total-Neg | -0.05 | 0.61*** | 0.25*** | -0.18* | 0.43*** | 0.71*** | -0.05 | 0.74*** | -0.49*** | -0.53*** | 0.45*** | 0.63*** | 0.66*** | -0.49*** | 0.58*** | -0.44*** | 0.56*** | -0.4*** | 0.96*** | -0.42*** | 0.79*** | 0.77*** | 0.78*** | - |  |  |  |  |  |  |  |  |
| 25. Pos-Matrix-End/ Total-Pos | 0.01 | -0.55*** | -0.12 | 0.34*** | -0.58*** | -0.73*** | 0.47*** | -0.58*** | 0.8*** | 0.47*** | -0.57*** | -0.68*** | -0.55*** | 0.73*** | -0.5*** | 0.64*** | -0.36*** | 0.6*** | -0.41*** | 0.97*** | -0.84*** | -0.78*** | -0.8*** | -0.38*** | - |  |  |  |  |  |  |  |
| 26. Negative Likert Endorsement Sum Bias | 0.01 | 0.7*** | 0.18* | -0.34*** | 0.63*** | 0.84*** | -0.29*** | 0.81*** | -0.71*** | -0.58*** | 0.59*** | 0.76*** | 0.78*** | -0.66*** | 0.64*** | -0.58*** | 0.56*** | -0.56*** | 0.81*** | -0.72*** | 0.91*** | 0.94*** | 0.93*** | 0.74*** | -0.75*** | - |  |  |  |  |  |  |
| 27. Positive Likert Endorsement Sum Bias | 0.1 | -0.66*** | -0.22** | 0.31*** | -0.64*** | -0.84*** | 0.34*** | -0.74*** | 0.74*** | 0.6*** | -0.58*** | -0.76*** | -0.68*** | 0.71*** | -0.63*** | 0.61*** | -0.5*** | 0.61*** | -0.69*** | 0.83*** | -0.91*** | -0.94*** | -0.96*** | -0.74*** | 0.78*** | -0.85*** | - |  |  |  |  |  |
| 28. Difference in Likert Endorsement Sum Bias | -0.04 | 0.71*** | 0.21** | -0.34*** | 0.66*** | 0.87*** | -0.32*** | 0.81*** | -0.75*** | -0.61*** | 0.61*** | 0.79*** | 0.76*** | -0.71*** | 0.66*** | -0.61*** | 0.56*** | -0.6*** | 0.79*** | -0.81*** | 0.95*** | 0.97*** | 0.98*** | 0.77*** | -0.79*** | 0.97*** | -0.96*** | - |  |  |  |  |
| 29. Neg-Likert-Sum/ Total-Neg | -0.01 | 0.62*** | 0.23** | -0.2** | 0.43*** | 0.71*** | -0.06 | 0.75*** | -0.5*** | -0.51*** | 0.46*** | 0.63*** | 0.69*** | -0.49*** | 0.57*** | -0.44*** | 0.57*** | -0.39*** | 0.99*** | -0.41*** | 0.81*** | 0.76*** | 0.76*** | 0.96*** | -0.41*** | 0.82*** | -0.69*** | 0.79*** | - |  |  |  |
| 30. Pos-Likert-Sum/ Total-Pos | 0.06 | -0.53*** | -0.12 | 0.31*** | -0.57*** | -0.7*** | 0.47*** | -0.55*** | 0.77*** | 0.46*** | -0.56*** | -0.66*** | -0.5*** | 0.72*** | -0.48*** | 0.63*** | -0.34*** | 0.58*** | -0.37*** | 0.98*** | -0.83*** | -0.75*** | -0.77*** | -0.37*** | 0.96*** | -0.68*** | 0.83*** | -0.78*** | -0.36*** | - |  |  |
| 31. Mean Negative Likert Bias | -0.01 | 0.62*** | 0.23** | -0.2** | 0.43*** | 0.71*** | -0.06 | 0.75*** | -0.5*** | -0.51*** | 0.46*** | 0.63*** | 0.69*** | -0.49*** | 0.57*** | -0.44*** | 0.57*** | -0.39*** | 0.99*** | -0.41*** | 0.81*** | 0.76*** | 0.76*** | 0.96*** | -0.41*** | 0.82*** | -0.69*** | 0.79*** | 1*** | -0.36*** | - |  |
| 32. Difference in Endorsement Bias | -0.06 | 0.39*** | 0.19** | -0.19* | 0.54*** | 0.64*** | -0.95*** | 0.34*** | -0.85*** | -0.2** | 0.58*** | 0.5*** | 0.39*** | -0.66*** | 0.32*** | -0.65*** | 0.21** | -0.7*** | 0.28*** | -0.63*** | 0.56*** | 0.56*** | 0.56*** | 0.29*** | -0.63*** | 0.53*** | -0.57*** | 0.57*** | 0.3*** | -0.63*** | 0.3*** | - |

*Note.* Abbreviations: QIDS-16-SR = Quick Inventory of Depressive Symptomatology; Neg-EndRec/Total-End = Proportion of Negative Endorsed and Recalled Words to Total Endorsed Words; Pos-EndRec/Total-End = Proportion of Positive Endorsed and Recalled Words to Total Endorsed Words; Neg-End/Total-Neg = Proportion of Negative Words Endorsed; Pos-End/Total-Pos = Proportion of Positive Words Endorsed; Neg-Likert-End/ Total-Neg = Proportion of Likert Negative Words Endorsed; Neg-Matrix-End/ Total-Neg = Proportion of Matrix Negative Words Endorsed; Pos-Matrix-End/ Total-Pos = Proportion of Matrix Positive Words Endorsed; Neg-Likert-Sum/ Total-Neg = Proportion of Likert Sum Negative Words; Pos-Likert-Sum/ Total-Pos = Proportion of Likert Sum Positive Words

* *p <*.05*, ** p <*.01*, *** p <* .001
